# The electroencephalogram in syncope

**DOI:** 10.1002/jgf2.177

**Published:** 2018-05-31

**Authors:** Junpei Komagamine

**Affiliations:** ^1^ Department of Internal Medicine National Hospital Organization Tochigi Medical Center Utsunomiya Tochigi Japan

To the Editor

I read with great interest the recent article reported by Izumi et al.^1^ The authors reported the findings of electroencephalograms (EEGs) of patients with cardiac syncope. In the discussion, they stated that few researchers have revealed the cause of systemic convulsion, which leads to whole‐brain ischemia measurable by EEG. However, the EEG findings during cardiac syncope were already investigated more than 50 years ago.^2^

One of the best‐known studies regarding the EEG findings during syncope was conducted by Gastaut et al.^2^, ^3^ They reported that during longer cardiac arrests (approximately 7–13 seconds), bilateral and synchronous slow waves often appeared but were not always accompanied by a loss of consciousness.^2^ They also reported that when cardiac arrest lasted more than around 14 seconds, one or two generalized clonic jerks appeared without affecting the EEG.^2^ The EEG in syncope can show two patterns: a slow‐flat‐slow pattern and a slow activity only pattern.^3^ The EEG findings of this case exhibit the latter pattern. Renowned neurologists have already clarified this issue.

## CONFLICT OF INTEREST

The authors have stated explicitly that there are no conflicts of interest in connection with this article.
